# RAFT-Mediated Polymerization-Induced Self-Assembly of Poly(Acrylic Acid)-*b*-Poly(Hexafluorobutyl Acrylate): Effect of the pH on the Synthesis of Self-Stabilized Particles

**DOI:** 10.3390/polym8060207

**Published:** 2016-05-27

**Authors:** Jianhua Zhou, Renyan He, Jianzhong Ma

**Affiliations:** 1 School of Light Industry Science and Engineering, Shaanxi University of Science and Technology, Xi’an 710021, China; renyanh@21cn.com; 2 Shaanxi Research Institute of Agricultural Products Processing Technology, Xi’an 710021, China

**Keywords:** RAFT polymerization, fluorine-containing amphiphilic block copolymer, polymerization-induced self-assembly

## Abstract

This paper describes a very simple strategy towards self-stabilized poly(acrylic acid)-*block*-poly(hexafluorobutyl acrylate) (PAA-*b*-PHFBA) block copolymer particles via reversible addition-fragmentation chain transfer (RAFT)-mediated polymerization-induced self-assembly. Hexafluorobutyl acrylate (HFBA) monomer conversion and number-average molar mass of PAA-*b*-PHFBA increased gradually with the increase in the pH value of the aqueous phase. When pH < 10, the molecular weight distributions of PAA-*b*-PHFBA were narrow, however, when the pH was raised to 11.55, PAA-*b*-PHFBA block copolymers had a broader distribution (*Đ*_M_ = 1.82) with a serious trailing toward the low molecular weight. Furthermore, the morphology and size of PAA-*b*-PHFBA latex particles were measured by transmission electron microscopy and dynamic light scattering. The results indicated that the PAA-*b*-PHFBA latex particles had a clear spherical core-shell structure and the latex particles’ size increased with the increase of pH value.

## 1. Introduction

Fluoropolymers have been widely studied and applied in many fields for their properties such as low surface energy, high resistance to thermal, chemical and weather attack, and low dielectric constant [1,2,3,4,5]. The fluorinated acrylate polymer with fluorinated side chains is a typical fluoropolymer [6], and has been widely used in textile finishing agents, anti-fouling or anti-corrosion coatings, construction protection coatings, and surface modifiers, *etc*. In these applications, the direct synthesis of fluorinated acrylate polymer latex is much favored. However, it is difficult to produce fluorinated acrylate polymer with a high content of fluorinated monomer via the traditional emulsion polymerization due to the extremely low solubility of fluorinated monomer in water. Some excellent works have been reported aiming at the efficient incorporation of fluoro-components into polymer latexes, such as the use of mini-emulsion [7] or microemulsion [8] polymerization techniques, and the introduction of fluoro-based surfactants [9], co-solvents [10], or phase transfer agents [11]. However, it has been noted that the fluoro-content in these latexes is still quite limited. In addition, the molecular structure and the distribution of fluorinated groups within the copolymers are generally incontrollable via traditional emulsion or mini-emulsion polymerization, and only the random copolymers can be obtained. An investigation on the molecular control of the fluorinated acrylate polymer would not only provide further optimization of their practical performance, but also meet the demands of the “high-tech” world, such as in considering optical, electronic, and biomedical fields.

Reversible-deactivation radical polymerization (RDRP) enables us to synthesize the block copolymers with well-defined molecular architecture and accurate molecular weight [12,13,14,15,16]. Among the available RDRP techniques, reversible addition-fragmentation chain transfer (RAFT) polymerization is one of the most effective and versatile methods for most monomers, and it is feasible to be carried out under heterophase conditions by using water as the continuous phase [17,18,19,20]. Poly(carboxylic acids) is a well-known water-soluble polymer widely used in industry for the production of superabsorbents, membranes, and coatings. Different kinds of poly(carboxylic acids) obtained by RAFT polymerization, for instance poly(acrylic acid) (PAA) and poly(methacrylic acid) (PMAA), have been widely applied in the emulsion polymerization [21,22,23]. PAA macro-RAFT agents have been used for the aqueous emulsion polymerization of hydrophobic monomers such as styrene (St) or *n*-butyl acrylate (BA) [24,25,26,27]. Both the colloidal stability and the molar masses could be under good control. More recently, Chenal *et al.* studied the batch emulsion polymerization of BA using PAA-RAFT of different chain lengths to form stable poly(butyl acrylate) (PBA) particles with long PBA blocks [28]. Chaduc *et al.* used PMAA-RAFT agents for the emulsion polymerization of polystyrene (PS) [29]. The study showed that the pH had a great impact on emulsion polymerization, and an increase in pH led to the decrease of monomer conversion, a broader molar mass distribution, and poorer controllability. It can be noticed that in all the above instances, many researchers focused on the aqueous emulsion polymerization of hydrophobic monomers such as styrene or butyl acrylate. The influence of some parameters on polymerization kinetics, emulsion stability, latex particle morphology, and size were investigated, such as poly(carboxylic acids) chain lengths, monomer concentration, and pH value, *etc*. However, the synthesis of fluorinated acrylate polymer via RAFT polymerization in the presence of poly(carboxylic acids) macro-RAFT agents has rarely been reported. Guo *et al.* synthesized poly(methacrylic acid)-*b*-poly(2,2,2-trifluoroethyl methacrylate) (PMAA-*b*-PTFEMA) in water/1,4-dioxane mixture solvent via one-pot two-step reaction protocol [30]. When the reaction was carried out under the solvent condition of 1,4-dioxane/water 1/4, the structure of PMAA-*b*-PTFEMA block copolymers was well controlled with narrow molar-mass dispersity.

In this research, fluorine-containing amphiphilic block copolymer poly(acrylic acid)-*block*-poly(hexafluorobutyl acrylate) (PAA-*b*-PHFBA) was synthesized by RAFT-mediated polymerization-induced self-assembly using poly(acrylic acid) both as a stabilizer and a RAFT agent in water. The influence of the pH value on the synthesis of PAA-*b*-PHFBA fluorine-containing amphiphilic block copolymer was systematically studied. The resulting fluorine-containing amphiphilic block copolymer was characterized by size exclusion chromatography (SEC), ^1^H-nuclear magnetic resonance (^1^H-NMR), transmission electron microscopy (TEM), and dynamic light scattering (DLS).

## 2. Materials and Methods

Acrylic acid (AA) was distilled under reduced pressure prior to use. Hexafluorobutyl acrylate (HFBA), supplied by XEOGIA Fluorine–Silicon Chemical Company (Harbin, China), was passed through an alkaline alumina chromatographic column before use. 4,4-azobis(4-cyanopentanoic acid) (ACPA) was supplied by Fluka Company (Buchs, Switzerland). *S*-1-dodecyl-*S*′-(α,α′-dimethyl-α″-acetic acid) trithiocarbonate (DDMAT) was synthesized and purified as described in [31].

### 2.1. Synthesis of Poly(Acrylic Acid) Macro-RAFT Agent

First, 2.0018 g (27.79 mmol) of AA, 252.8 mg (0.66 mmol) DDMAT, 19.9 mg (0.07 mmol) of ACPA, and 10.00 mL of ethanol were added into a 100 mL three-necked round-bottom flask equipped with a magnetic stirrer, a condenser, and an argon inlet and outlet. The mixture was deoxygenated with argon gas for 30 min, and then immersed in a heated water bath at 80 °C. The reaction went on for 6 h and ended by inserting the flask in an ice bath. The solvent was driven off using a rotary evaporator and the unreacted monomers were removed by precipitation of the polymer into cold *n*-hexane. Thus, the yellow powder poly(acrylic acid) macro-RAFT agent was obtained.

### 2.2. RAFT Emulsion Polymerization of Hexafluorobutyl Acrylate Mediated by Poly(Acrylic Acid) Macro-RAFT Agent

The recipe for RAFT emulsion polymerization of hexafluorobutyl acrylate is described in Table 1. For a typical reaction procedure (Latex 5 in Table 1), 240.5 mg (0.10 mmol) of PAA_27_-RAFT and 210.1 mg (5.25 mmol) of NaOH were dissolved in 6.01 mL of deionized water in a 100 mL flask. Then, 529.1 mg (2.24 mmol) of HFBA was added to the aqueous solution, and the mixture was stirred at room temperature for 30 min. Then, 1 mL of an aqueous solution of ACPA (concentration = 1.20 mol·L^−1^, neutralized by 3.5 mol equiv of NaHCO_3_) was added to the reaction mixture. The mixture was deoxygenated with argon gas for 30 min, and then was kept at 80 °C for 4 h. Finally, the reaction ended by inserting the flask in an ice bath. The structure of PAA-*b*-PHFBA was confirmed by ^1^H-NMR and size exclusion chromatography (SEC) after demulsifying the latexes and drying them under a vacuum freeze drier to remove the unreacted monomers and water.

### 2.3. ^1^H-Nuclear Magnetic Resonance (^1^H-NMR)

^1^H-NMR spectra were recorded on a 400 MHz Advance III spectrometer (Bruker, Rheinstetten, Germany) using DMSO-d_6_ or D_2_O as solvent.

### 2.4. Size Exclusion Chromatography (SEC)

The molecular weights and molecular weight distributions of PAA and PAA-*b*-PHFBA were determined on a Waters e2695 size exclusion chromatography (SEC, Waters, Milford, MA, USA) equipped with a refractive index detector. PAA was analyzed with a SEC apparatus running in 0.1 mol·L^−1^ sodium nitrate aqueous solution at a flow rate of 0.8 mL·min^−1^. The column system was calibrated by monodispersed linear polyethylene glycol standards. PAA-*b*-PHFBA was analyzed with a SEC apparatus running in tetrahydrofuran (THF) at a flow rate of 1.0 mL·min^−1^. The column system was calibrated by monodispersed linear polystyrene standards. Whatever SEC apparatus was used, all samples were injected with a concentration of 3 mg·mL^−1^ after filtration through a 0.45 μm pore-size membrane.

### 2.5. Dynamic Light Scattering (DLS)

The synthesized emulsion was diluted to a concentration of 0.1%. The latex particle size was measured with a Malvern Nano ZS instrument (Malvern Instruments, Worcestershire, UK) at a fixed scattering angle of 90° at room temperature.

### 2.6. Transmission Electron Microscopy (TEM)

Transmission electron microscopy (TEM) micrographs of the PAA-*b*-PHFBA block copolymers dispersions were observed on a FEI Tecnai G2 F20 S-TWIN electron microscope (FEI Company, Hillsboro, OR, USA) at a voltage of 200 kV. The sample was stained with 1.5% phosphotungstic acid solution.

### 2.7. pH Monitoring

The pH value of the aqueous phase was probed by a pH-meter (PHS-25) using an E-201-C pH composite electrode.

## 3. Results

### 3.1. Synthesis of Poly(Acrylic Acid) Macro-RAFT Agent by Solution Polymerization in Ethanol

**Scheme 1:**
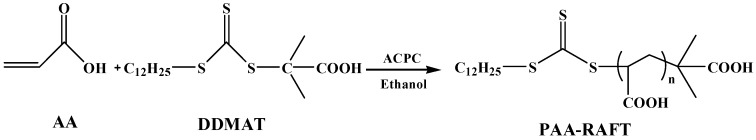
Synthesis of PAA macro-RAFT agent by RAFT polymerization.

**Figure 1:**
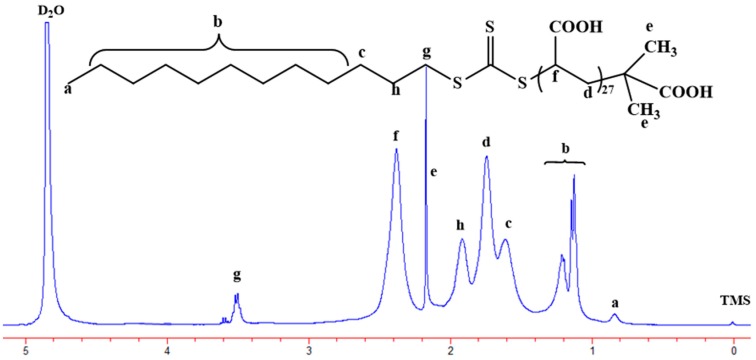
^1^H-NMR spectrum of PAA macro-RAFT agent.

**Figure 2:**
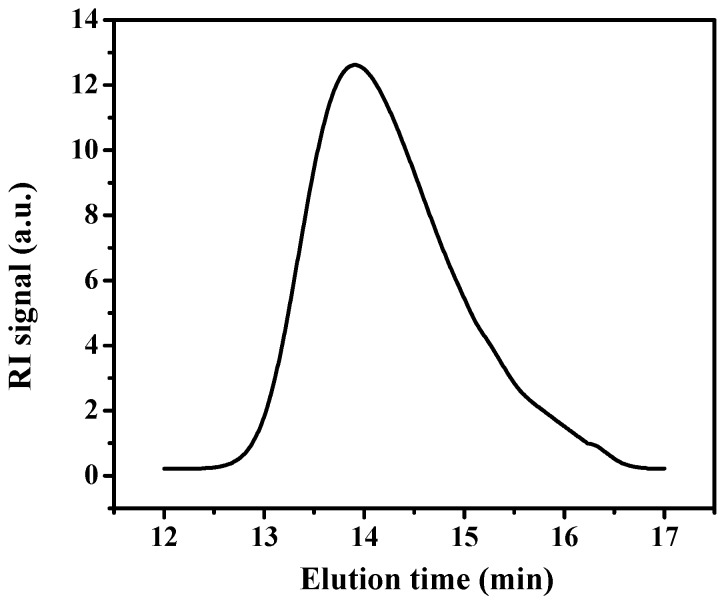
SEC trace of PAA macro-RAFT agent.

Poly(acrylic acid) (PAA) macro-RAFT agent was synthesized by RAFT polymerization using *S*-1-dodecyl-*S*′-(α,α′-dimethyl-α″-acetic acid) trithiocarbonate (DDMAT) as chain transfer agent in ethanol. The polymerization process is illuminated in Scheme 1. The structure of PAA macro-RAFT agent was characterized by ^1^H-NMR spectroscopy and SEC. The ^1^H-NMR spectrum of PAA macro-RAFT agent dissolved in D_2_O is illustrated in Figure 1. Feature signals of the PAA segment were apparent (δ = 2.44 ppm (–CH(COOH)CH_2_–), δ = 1.3–1.8 ppm (–CH(COOH)CH_2_–)). The signals at 0.90 and 1.26 ppm are attributed to the methyl protons and methylene protons of DDMAT RAFT agent, respectively, and this provides good evidence that the PAA macro-RAFT agent has the end derived from the original RAFT agent, indicating that PAA is able to act as a macro-RAFT agent and be extended through further polymerization. The molecular weight and composition of the PAA macro-RAFT agent could be calculated by comparing the intensity of methine protons (H_f_) of PAA at 2.44 ppm to the intensity of methyl protons (H_a_) of –C_12_H_25_ chain moiety at 0.90 ppm. Therefore, the *M*_n_ obtained from ^1^H-NMR analysis is 2.30 × 10^3^ g/mol, and the PAA macro-RAFT agent has 27 acrylic acid units. Furthermore, the molecular weight of the PAA macro-RAFT agent and its distribution were characterized by SEC. The *M*_n_ of the PAA macro-RAFT agent is 7.76 × 10^3^ g/mol and the molar-mass dispersity is 1.21 (Figure 2). However, the SEC curve of the PAA macro-RAFT agent shows a tailing toward the low molecular weight, which is attributed to the absorption of PAA onto the SEC column. The *M*_n_ determined by SEC is higher than that calculated using ^1^H-NMR. This phenomenon may be explained by the fact that the acrylic acid groups may further complicate the issue through interactions with the column and themselves, although these interactions should largely be screened by the sodium nitrate in the eluent. On the other hand, there is a large difference in the hydrodynamic volume of PAA and polyethylene glycol standard having the same molecular weight. The low molecular weight distribution of the PAA macro-RAFT agent confirms the good control of the RAFT technique.

### 3.2. RAFT Emulsion Polymerization of Hexafluorobutyl Acrylate Using PAA Macro-RAFT Agent

**Scheme 2:**
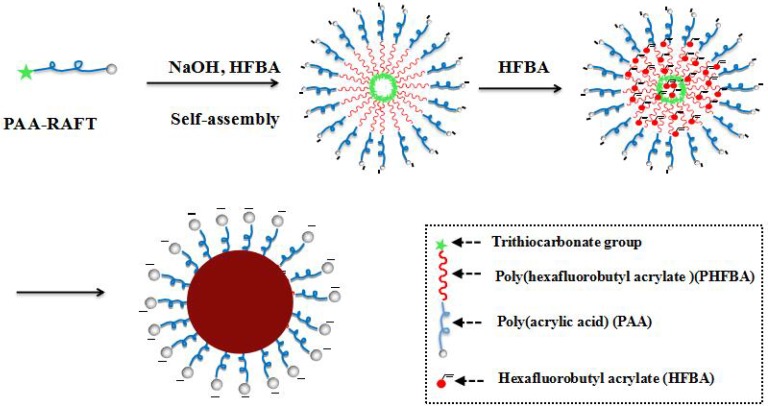
Reaction scheme for the synthesis of poly(acrylic acid)-*b*-poly(hexafluorobutyl acrylate) with PAA macro-RAFT agent as stabilizer.

The PAA macro-RAFT agent is a living polymer which contains a hydrophilic PAA segment and trithiocarbonate end group [32]. The PAA macro-RAFT agent can be further chain-extended with hexafluorobutyl acrylate (HFBA) in water to form amphiphilic block copolymers able to self-assemble into micelles in which the polymerization continues, as shown in Scheme 2. This approach has evolved as a simple way of producing amphiphilic block copolymer particles by polymerization-induced self-assembly (PISA) performed in water [33,34,35,36,37,38,39,40,41,42,43,44,45].

The aqueous RAFT-mediated emulsion polymerization performed with polyacid-based marco-RAFT agents has been reported to be very sensitive to the pH of the reaction medium [46,47,48,49]. In order to study the influence of the pH value on the emulsion polymerization of HFBA mediated by a PAA macro-RAFT agent, a series of emulsion polymerization experiments were carried out in aqueous solutions with different pH values (see Table 1).

**Figure 3:**
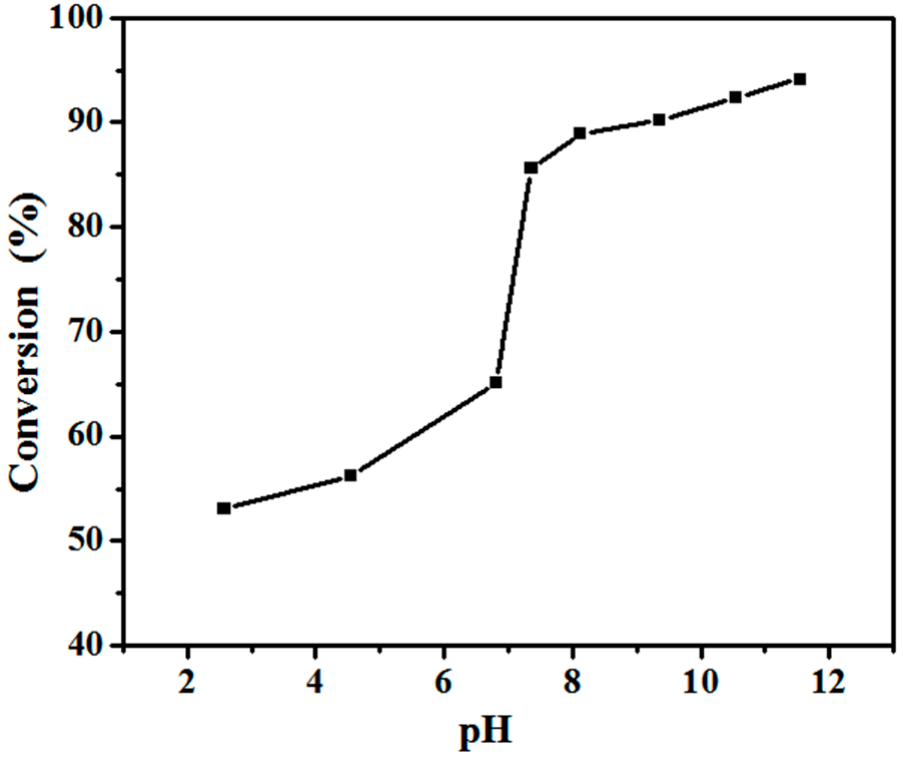
Synthesis of PAA-*b*-PHFBA block copolymer with PAA macro-RAFT agent at different pH values. Curve of conversion *vs.* different pH values.

The pH value has a great impact on the HFBA conversion, as shown in Figure 3. The monomer conversion increases gradually with the increase in the pH value of the aqueous phase. Under acidic conditions (pH < 6.82), the monomer conversion is limited to less than 65% after 210 min of polymerization time. Additionally, the resultant colloids synthesized under acidic conditions were not stable, and more or less an oily layer of unreacted HFBA monomers floated on the surface of emulsion. This can be ascribed to the poor dispersion of the PAA macro-RAFT agent in water due to the incomplete ionization of the carboxylic groups of the PAA macro-RAFT agent under acidic conditions because the pKa of PAA is about 5.5 [50]. However, it was found that the monomer conversion increases substantially when the emulsion RAFT polymerization is conducted under alkaline conditions. For example, the monomer conversion reaches 86% in 210 min at pH = 7.36. This phenomenon stems from the pronounced dissociation of carboxylic groups in the PAA macro-RAFT agent. At high pH value, the carboxyl groups on the PAA macro-RAFT agent are almost completely ionized, and therefore less molecules of PAA macro-RAFT agent are needed to construct single surfactant micelle. That is to say, with the increase of pH, the number of polymer micelles as a principal polymerization locus increases, thereby resulting in the increase of the monomer conversion.

**Figure 4:**
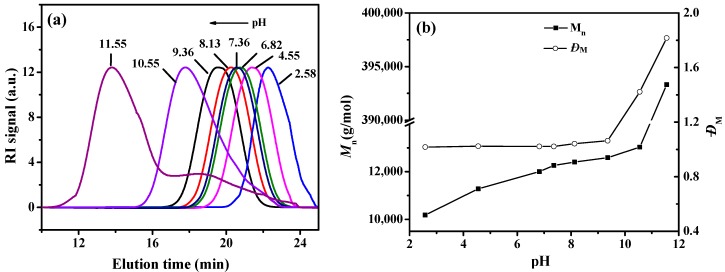
SEC analysis for PAA-*b*-PHFBA block copolymers prepared by RAFT emulsion polymerization of hexafluorobutyl acrylate in the presence of PAA macro-RAFT agent at different pH values. (**a**) Evolution of SEC curves with different pH values; (**b**) Dependence of number-average molar mass (*M*_n_) and molar-mass dispersity (*Đ*_M_) on pH value.

Figure 4 is the SEC chromatogram of the PAA-*b*-PHFBA block copolymers prepared by RAFT emulsion polymerization of hexafluorobutyl acrylate in the presence of the PAA macro-RAFT agent at different pH values. As shown in Figure 4a, the SEC curve shifts to a higher molecular weight region with the increase of pH. The change trend of *M*_n_ *versus* pH value is consistent with that of conversion *versus* pH value. Molar-mass dispersity of the PAA-*b*-PHFBA block copolymers is depicted in Figure 4b. It can be observed that the copolymers have a relatively narrow molar-mass dispersity (*Đ*_M_ < 1.07) for pH < 10. However, when the pH is raised to 11.55, the molecular weight (*M*_n_) is approximately an order of magnitude higher than that at pH < 10, and the SEC chromatogram shows the PAA-*b*-PHFBA block copolymer has a broader molar-mass dispersity (*Đ*_M_ = 1.82) with a serious trailing toward the low molecular weight. A possible explanation might be found in the serious hydrolysis of the trithiocarbonate C–S bond [49,51], resulting in the bad polymerization control for the polymerization of HFBA at pH 11.55.

**Figure 5:**
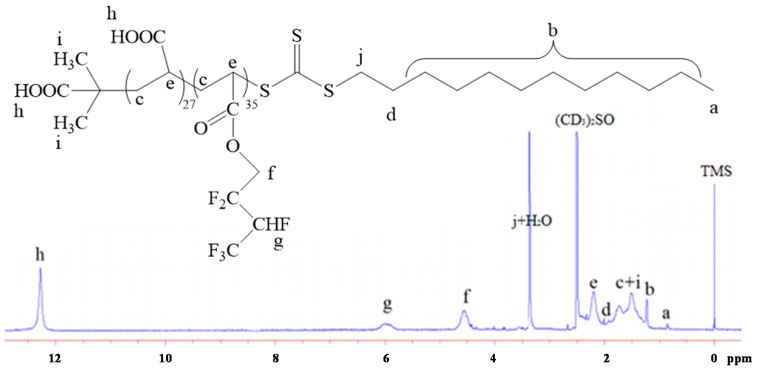
^1^H-NMR spectrum of PAA-*b*-PHFBA.

The structure of the PAA-*b*-PHFBA copolymer was confirmed by ^1^H-NMR spectroscopy. As shown in Figure 5, feature signals of the PAA segment were apparent (δ = 2.18 ppm (–CH(COOH)CH_2_–), δ = 1.3–1.8 ppm (–CH(COOH)CH_2_–)), and PHFBA segment (δ = 5.98 ppm (CF_3_CHFCF_2_CH_2_–), δ = 4.55 ppm (CF_3_CHFCF_2_CH_2_–)). The signals at 0.86 and 1.23 ppm are attributed to the methyl protons and methylene protons of RAFT agent. The *M*_n_ obtained from the ^1^H-NMR analysis is 8.15 × 10^3^ g/mol, and the PAA-*b*-PHFBA has 27 acrylic acid units and 35 HFBA units. The ^1^H-NMR result demonstrates the successful preparation of the PAA-*b*-PHFBA block copolymer.

### 3.3. Morphology and Size of PAA-b-PHFBA Latex Particles

**Figure 6:**
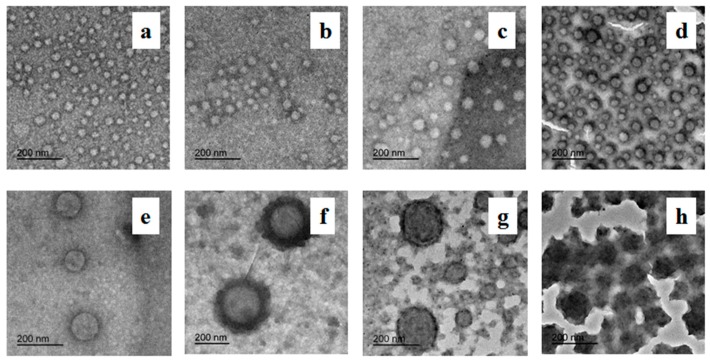
TEM micrographs of PAA-*b*-PHFBA latex particles obtained at different pH values: pH = 2.58 (**a**); pH = 4.55 (**b**); pH = 6.82 (**c**); pH = 7.36 (**d**); pH = 8.13 (**e**); pH = 9.36 (**f**); pH = 10.55 (**g**); and pH = 11.55 (**h**).

The morphology and size of the PAA-*b*-PHFBA latex particles was measured by TEM and DLS. Figure 6 shows TEM images of the polymer latex particles. With careful staining of the particles using 1.5% phosphotungstic acid, the spherical core-shell latex particles can be observed due to the difference of electron penetrability of the core and shell [52,53]. The light and the dark regions in the particles correspond to the PHFBA core and PAA shell, respectively. For pH < 10, the latex particle size increases from 54 to 325 nm, and the shell thickness increases from 5 to 38 nm with the increase of pH from 2.58 to 9.36. Assuming a fully extended conformation of polymer and a monomer contour length of 0.24 nm [54], the contour lengths of the PAA-*b*-PHFBA polymers calculated from the molecular weights obtained by SEC would vary from 26 to 29 nm with the increase of pH from 2.58 to 9.36. This indicates the contour lengths of the PAA-*b*-PHFBA polymers are smaller than the radii of latex particles measured by TEM. Therefore, it appears that the coalescence of smaller primary particles must occur in the polymerization process to form the bigger particles with a better colloidal stability. The increase of the latex particle size with increasing pH is related to the critical molar mass that the hydrophobic block has to reach for the corresponding block copolymer to start to self-assemble and induce nucleation. When the pH increases, charged PAA blocks become more hydrophilic and the molar mass of the PHFBA block needs to be higher for the block copolymers to become amphiphilic. In addition, the carboxyl groups on the PAA blocks are ionized gradually with the increase of pH value, and the PAA chains are further extended with exposure to a high pH aqueous solution. For pH > 10, two ranges of particle size are obtained: small and rather high-number particles (D_n_ around 70 nm), and bigger and rather low-number particles (D_n_ around 200 nm). A possible explanation might be found in the serious hydrolysis of the trithiocarbonate C–S bond at such high pH [48,55], which results in the loss of polymerization control and broad particle size distributions.

**Figure 7:**
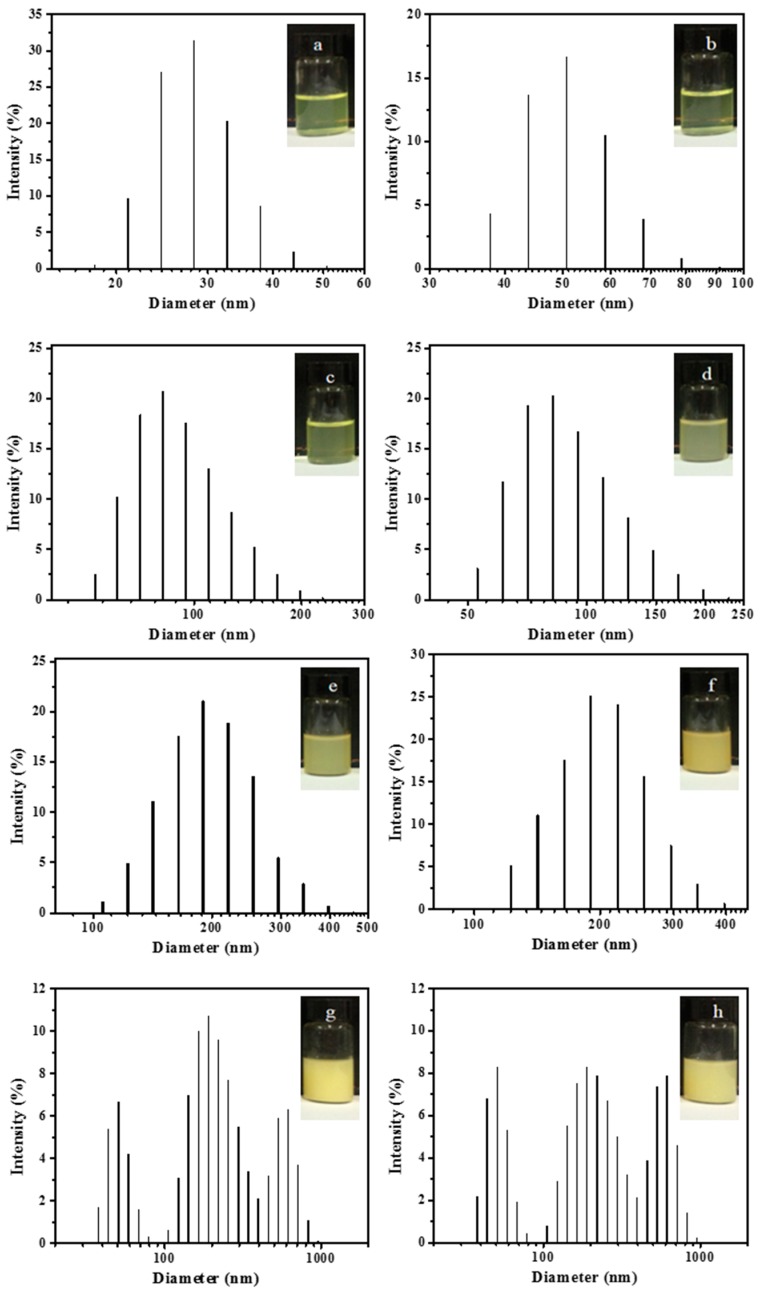
Particle size distributions determined by DLS of the PAA-*b*-PHFBA colloid particles with different pH values: pH = 2.58 (**a**); pH = 4.55 (**b**); pH = 6.82 (**c**); pH = 7.36 (**d**); pH = 8.13 (**e**); pH = 9.36 (**f**); pH = 10.55 (**g**) and pH = 11.55 (**h**).

Figure 7 shows DLS graphs of the size distribution of the PAA-*b*-PHFBA colloid particle with different pH values. For pH < 10, the average diameter of the PAA-*b*-PHFBA block copolymer latex particle increases from 28 to 220 nm. Whatever the pH is, no precipitation occurs after six months. However, above pH = 10, the average particle size of the PAA-*b*-PHFBA colloid particle (598 nm for pH = 10.55, 606 nm for pH = 11.55) is much higher than that for pH < 10, emulsion appearance is more cloudy, and some precipitation can be found immediately after the reaction was stopped. The change trend of emulsion particle size *versus* pH value observed with DLS is consistent with the results measured by TEM. According to the results of TEM and DLS analysis mentioned above, it can be confirmed that the spherical core-shell PAA-*b*-PHFBA colloid particles have been successfully obtained by RAFT emulsion polymerization.

## 4. Conclusions

Poly(acrylic acid)-*b*-poly(hexafluorobutyl acrylate) (PAA-*b*-PHFBA) block copolymer was produced by RAFT-mediated polymerization-induced self-assembly in water using a poly(acrylic acid) macro-RAFT agent. First, a well-defined poly(acrylic acid) macro-RAFT agent was obtained using *S*-1-dodecyl-*S*′-(α,α′-dimethyl-α″-acetic acid) trithiocarbonate as a RAFT agent in ethanol. The poly(acrylic acid) macro-RAFT agent was then chain-extended *in situ* with hexafluorobutyl acrylate (HFBA) to form a PAA-*b*-PHFBA block copolymer chains of controlled molar mass that self-assemble into latex particles. The HFBA conversion increased gradually with the increase of pH value. The change trend of *M*_n_ *versus* pH value was consistent with that of conversion *versus* pH value. When pH < 10, the molecular weight distributions (*Đ*_M_ < 1.03) of the PAA-*b*-PHFBA block copolymers were narrow. However, PAA-*b*-PHFBA block copolymers had the broader distribution (*Đ*_M_ > 1.43) for pH > 10. TEM and DLS investigations showed spherical core-shell latex particles composed of PAA-*b*-PHFBA block copolymers were formed. The latex particle size increased with the increase of pH value. Only two ranges of particle size were observed for pH > 10.

## Figures and Tables

**Table 1:** Experimental conditions and results for reversible addition-fragmentation chain transfer (RAFT) emulsion polymerization of hexafluorobutyl acrylate in water.

Sample ^a^	pH	Conversion ^b^ (%)	*M*_n_^c^ (g·mol^−1^)	*Đ*_M_^c^
Latex 1	2.58	53.12	1.02 × 10^4^	1.017
Latex 2	4.55	56.24	1.13 × 10^4^	1.023
Latex 3	6.82	65.12	1.20 × 10^4^	1.021
Latex 4	7.36	85.61	1.23 × 10^4^	1.021
Latex 5	8.13	89.92	1.24 × 10^4^	1.021
Latex 6	9.36	90.23	1.26 × 10^4^	1.024
Latex 7	10.55	92.32	1.30 × 10^4^	1.423
Latex 8	11.55	94.12	3.93 × 10^5^	1.817

^a^ All the experiments were performed at *T* = 80 °C for 5 h with [HFBA]/[PAA_27_-RAFT] = 70 and [PAA_27_-RAFT] = 7.56 × 10^−3^ mmol·L^−1^; ^b^ Monomer conversion determined by gravimetric analysis; ^c^ Experimental number-average molar mass (*M*_n_) and molar-mass dispersity (*Đ*_M_) of the block copolymers determined by SEC in THF, 1.0 mL·min^−1^, calibrated using linear polystyrene standards. PAA, Poly(acrylic acid); HFBA, hexafluorobutyl acrylate; SEC, size exclusion chromatography; THF, tetrahydrofuran.
