# Erratum: RAFT-Mediated Polymerization-Induced Self-Assembly of Poly(Acrylic Acid)-*b*-Poly(Hexafluorobutyl Acrylate): Effect of the pH on the Synthesis of Self-Stabilized Particles. *Polymers*, 2016, *8*, 207

**DOI:** 10.3390/polym8110384

**Published:** 2016-11-01

**Authors:** Jianhua Zhou, Renyan He, Jianzhong Ma

**Affiliations:** 1 School of Light Industry Science and Engineering, Shaanxi University of Science and Technology, Xi’an 710021, China; renyanh@21cn.com; 2 Shaanxi Research Institute of Agricultural Products Processing Technology, Xi’an 710021, China

The authors wish to make a change to the published paper [1]. Scheme 2 should be replaced with that shown below. The authors apologize for any inconvenience caused.

The change does not affect the scientific results. The manuscript will be updated and the original will remain online on the article webpage http://www.mdpi.com/2073-4360/8/6/207.

## Figures and Tables

**Scheme 2:**
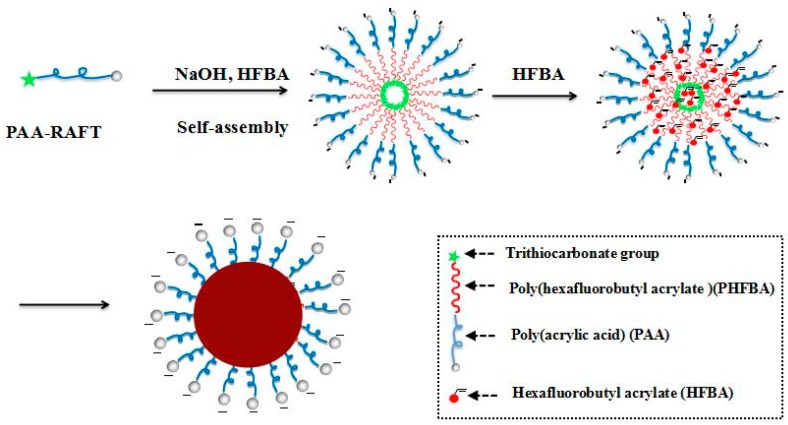
Reaction scheme for the synthesis of poly(acrylic acid)-*b*-poly(hexafluorobutyl acrylate) with PAA macro-RAFT agent as stabilizer.
